# Obesity and the cost of living crisis

**DOI:** 10.1038/s41366-022-01242-9

**Published:** 2022-12-02

**Authors:** Eric Robinson

**Affiliations:** Department of Psychology, Eleanor Rathbone Building, University of Liverpool, Liverpool, L69 7ZA UK

**Keywords:** Public health, Risk factors

2022 has seen the emergence of a global cost of living crisis driven by rapid increases in the cost of energy and food. The result will be a growing number of families experiencing short-term financial turmoil and long-term financial hardship [1]. Increasing inflation results in households with proportionately less disposable income, and this will invariably impact on food purchasing. One likely impact will be the tipping of many people from lesser economically developed countries into extreme poverty and starvation [2]. Furthermore, in the context of an already well-established obesity crisis, the cost of living crisis may also create the perfect storm for driving global obesity prevalence further upwards.

Families already have to choose between cheap and readily available energy-dense foods vs. more costly healthier food options, often financially and also in terms of preparation time. As financial hardship hits, choosing the latter will become more difficult. Households with the lowest incomes are less able to place long-term health at the top of their considerations when buying, choosing and cooking food. Recent research suggests that this is one likely reason why lower socioeconomic status is associated with higher BMI [3]. If left unchecked, the cost of living crisis has the potential to further widen socioeconomic inequalities in obesity by disproportionately affecting disadvantaged families and communities already at risk of obesity.

The stress of financial crises is thought to damage mental health, and the well-being of many of those experiencing financial hardship will be at risk [4]. Poorer mental health likely decreases motivation in the context of obesity management [5] and is a risk factor for increased weight gain among the general population, potentially through biopsychological mechanisms such as comfort eating [6]. Although research is less convincing and comprehensive in humans than in non-human animals, resource deprivation and insecurity may directly impact on biological systems to increase fat deposition and weight gain [7]. Well before the emergence of the current cost of living crisis, research had documented the concerning number of people worldwide living in food insecurity. Experiencing food insecurity is a risk factor for obesity and other health problems [8]. The cost of living crisis therefore has potential to move many more into food insecurity and further increase obesity among those already experiencing financial hardship.

Obesity research in the context of the current and future financial crises will have both theoretical and applied value. The COVID-19 pandemic stimulated a large amount of research into understanding how obesity increased risk of death, how those living with obesity were disproportionately affected, and the impact the pandemic has had on obesity prevalence [9]. The current cost of living crisis provides an (unfortunate) opportunity to study how and why diet, physical activity and obesity are affected in those experiencing acute financial hardship. Furthermore, documenting the impacts that the cost of living crisis has on absolute and relative inequalities in obesity prevalence will be important.

How countries respond to the cost of living crisis will matter, and invariably will differ from one government to another. Although there will be universal efforts to address inflation and financial hardship, in the context of obesity the devil will be in the detail. In recent years the UK government have implemented and proposed the introduction of a range of population-level anti-obesity measures including, among others, banning of price promotions and advertisement of unhealthy food products. However, in response to the cost of living crisis, there are suggestions that government will reverse the introduction of such measures in order to remove constraints on businesses and drive promote economic growth [10].

If governments deprioritise obesity policy to instead try and spur short-term economic growth, not only will obesity be worsened, but it is likely that there be damaging longer-term economic impacts. The current global burden of obesity is large and will continue to grow if upwards obesity prevalence trends continue. Obesity policy in many countries has historically been fragmented and not been considered in the wider context of other major societal challenges, such as climate change and financial crises. If this continues, then the obesity crisis will be with us for a very long time or even worse, indefinitely.
